# Objective Measurements of Physical Activity and Sedentary Behavior Using Wearable Devices in Patients With Axial Spondyloarthritis: Protocol for a Systematic Review

**DOI:** 10.2196/23359

**Published:** 2021-11-25

**Authors:** Thomas Carlin, Julie Soulard, Timothée Aubourg, Johannes Knitza, Nicolas Vuillerme

**Affiliations:** 1 AGEIS Université Grenoble Alpes Grenoble France; 2 LabCom Telecom4Health Orange Labs and Université Grenoble Alpes, CNRS, Inria, Grenoble INP-UGA Grenoble France; 3 Grenoble Alpes University Hospital Grenoble France; 4 Department of Internal Medicine 3 - Rheumatology and Immunology Friedrich-Alexander University Erlangen-Nürnberg Erlangen Germany; 5 Institut Universitaire de France Paris France

**Keywords:** axial spondyloarthritis, rheumatology, physical activity, sedentary behavior, objective measures, wearable, systematic review

## Abstract

**Background:**

Axial spondyloarthritis (axSpA) is a subgroup of inflammatory rheumatic diseases. Practicing regular exercise is critical to manage pain and stiffness, reduce disease activity, and improve physical functioning, spinal mobility, and cardiorespiratory function. Accordingly, monitoring physical activity and sedentary behavior in patients with axSpA is relevant for clinical outcomes and disease management.

**Objective:**

This review aims to determine which wearable devices, assessment methods, and associated metrics are commonly used to quantify physical activity or sedentary behavior in patients with axSpA.

**Methods:**

The PubMed, Physiotherapy Evidence Database (PEDro), and Cochrane electronic databases will be searched, with no limit on publication date, to identify all the studies matching the inclusion criteria. Only original English-language articles published in a peer-reviewed journal will be included. The search strategy will include a combination of keywords related to the study population, wearable devices, physical activity, and sedentary behavior. We will use the Boolean operators “AND” and “OR” to combine keywords as well as Medical Subject Headings terms.

**Results:**

Search strategy was completed in June 2020 with 23 records obtained. Data extraction and synthesis are currently ongoing. Dissemination of study results in peer-reviewed journals is expected at the end of 2021.

**Conclusions:**

This review will provide a comprehensive and detailed synthesis of published studies that examine the use of wearable devices for objective assessment of physical activity and sedentary behavior in patients with axSpA.

**Trial Registration:**

PROSPERO CRD42020182398; https://www.crd.york.ac.uk/prospero/display_record.php?RecordID=182398

**International Registered Report Identifier (IRRID):**

PRR1-10.2196/23359

## Introduction

Axial spondyloarthritis (axSpA) is a subgroup of inflammatory rheumatic diseases that predominantly affect the axial skeleton and the sacroiliac joints [1-3]. Some clinical manifestations of axSpA are inflammatory back pain and spinal stiffness [4-7]. It is now well established that practicing regular exercise results in health benefits in patients with axSpA by managing correctly inflammatory joint diseases [8-10], pain [11], and stiffness [11]; by decreasing disease activity [12]; and by improving physical functioning [13,14], spinal mobility [14,15], and cardiorespiratory function [16]. Interestingly, lack of exercise has been identified as a risk factor for the appearance of depressive symptoms [17], suggesting the benefits of practicing physical activity to manage mental comorbidities of axSpA [17].

Accordingly, monitoring physical activity and sedentary behavior in patients with axSpA is relevant for clinical outcomes and disease management. Traditionally, physical activity and sedentary behavior are assessed using either subjective measures (eg, self-reported questionnaires) [18-27] or objective measures (eg, accelerometers) [23-26,28-31]. At this point, however, recent studies have demonstrated that self-reported physical activity is a less valid and less reliable method for measuring physical activity in patients with axSpA than device-measured physical activity [32,33]. These results support the use of wearable devices for objective assessment of physical activity or sedentary behavior in patients with axSpA [14,32,34-38] as well as in patients with rheumatoid arthritis (eg, [34]). At this point, however, to the best of our knowledge, no systematic review has identified and synthesized the available evidence on the use of wearable devices for this specific population. This review is hence specifically designed to address this issue and, more specifically, to answer the following question: which wearable devices, assessment methods, and associated metrics have been used to quantify physical activity or sedentary behavior in patients with axSpA?

## Methods

### Design

This systematic review will be conducted under the PRISMA (Preferred Reporting Items for Systematic Reviews and Meta-Analyses) guidelines provided by Moher et al [39].

The present review protocol has been registered in PROSPERO (CRD42020182398). This research is exempt from ethics approval because the data are available through published and publicly available resources.

### Inclusion Criteria

All published studies assessing physical activity or sedentary behavior patterns of adults with a diagnosis of axSpA will be included. Only original articles published in English in a peer-reviewed scientific journal will be covered by this review.

The following inclusion criteria will be used:

- Type of participants: All articles including participants aged over 18 years with a diagnosis of axSpA will be included.
- Type of intervention or exposure: Participants are not required to undergo any type of intervention. This review is designed to identify and synthesize current practices of wearable-based monitoring of physical activity or sedentary behavior in patients with axSpA. The effects of the interventions will not be analyzed. Only baseline data on physical activity and sedentary will be extracted.
- Type of outcome measurements: Studies will discuss the use of wearable devices to measure physical activity or sedentary behavior in patients with axSpA.

### Exclusion Criteria

Case reports, abstracts, editorials, conference abstracts, letters to the editor, reviews, and meta-analyses will be excluded from this review. Furthermore, published studies without an objective assessment of physical activity or sedentary behavior of patients with axSpA extracted from wearable devices will be ineligible.

### Data Sources and Search Strategy

The following electronic databases will be systematically searched to identify studies satisfying the search criteria: PubMed, Physiotherapy Evidence Database (PEDro), and Cochrane, with no limit on publication date.

The search strategy includes a combination of the following keywords, using the Boolean operators “AND” and “OR” and Medical Subject Headings terms:

(“ankylosing spondylitis” OR spondyloarthritis) AND (“wearable technology” OR “wearable sensor” OR “wearable device” OR “ambulatory monitoring” OR “fitness tracker” OR “activity tracker” OR “activity monitor” OR “step counter” OR Actigraphy OR Pedometer OR “inertial sensor” OR “inertial measurement unit” OR “pendant sensor” OR accelerometer OR inclinometer OR gyroscope) AND (“physical activity” OR “physical activities” OR “physical inactivity” or “physical exertion” OR fitness OR exercise OR sports OR lifetime OR training OR “leisure time” OR “aerobic activity” OR “aerobic activities” OR “activity level” OR Sedentary OR Sedentariness OR “sedentary behaviour” OR “sedentary behaviours” OR “sedentary behavior” OR “sedentary behaviors” OR “sedentary time” OR “sedentary lifestyle” OR “sedentary activity” OR “sedentary activities” OR “prolonged sitting” OR “sitting time” OR seated OR Standing OR walking OR running OR sleep OR Step OR steps OR “covered distance” OR Vo2 or “Maximal oxygen uptake” OR “energy expenditure” OR “moderate- to vigorous-intensity physical activity” OR MVPA).

### Study Selection

Two reviewers (A and B) will independently screen each search match and decide on their potential inclusion based on data extracted from titles, abstracts, and keywords. Subsequently, the full-length text of the potentially included studies will be reviewed in detail to determine whether they satisfy the inclusion criteria mentioned above. Finally, based on inclusion and exclusion criteria, each reviewer (A and B) will decide independently on the eligibility of each search match. Any discrepancies between the two reviewers (A and B) will be resolved at a consensus meeting. If disagreement persists, a third reviewer (C) will be consulted to reach a final decision.

### Risk of Bias in Individual Studies

As our aim is not to evaluate the effect of an intervention, we will not perform a risk of bias assessment. We will conduct a systematic review of published studies that used wearable sensors for objective assessment of physical activity or sedentary behavior in patients with axSpA.

### Data Extraction

**Figure 1:**
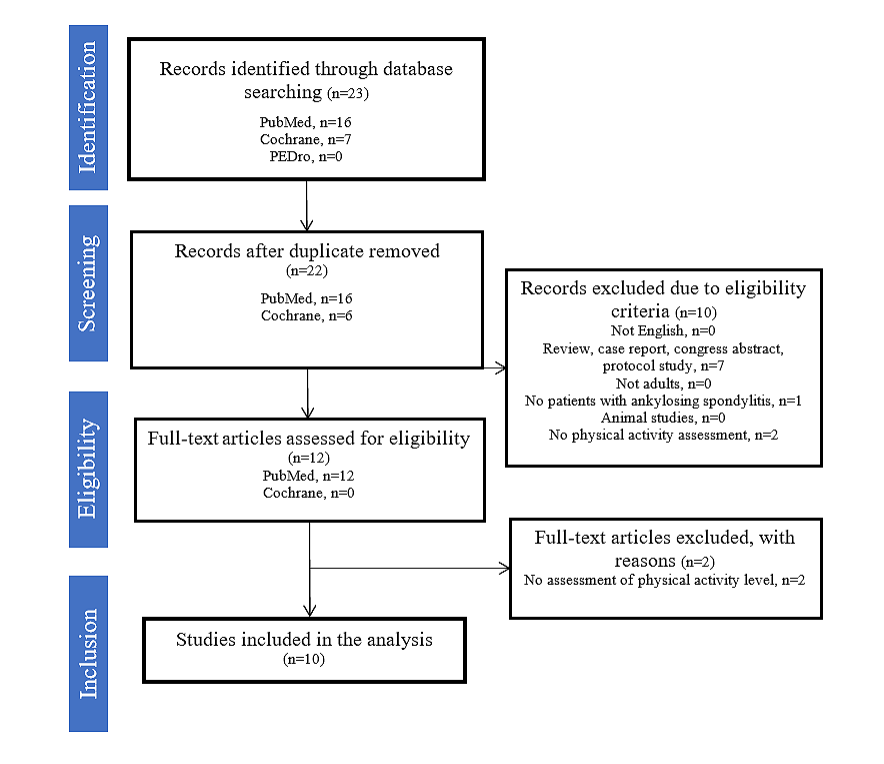
PRISMA (Preferred Reporting Items for Systematic Reviews and Meta-Analyses) flow chart of the selection process. PEDro: Physiotherapy Evidence Database.

In line with PRISMA guidelines [39], a flow chart summarizes each stage of the review with the corresponding number of citations (Figure 1).

Furthermore, the following 4 data sets will be extracted from the retrieved articles by two independent reviewers:

1. Study characteristics: first author, title, year of publication, journal’s name, country, study design, study duration, mention of any adverse events that occurred during the study, and funding.
2. Sample description: sample size, age, gender, weight, height, body mass index, health status, disease duration, functional status measurements, level of pain, description of radiographic damage, biologic medications, Bath Ankylosing Spondylitis Functional Index (BASFI), Bath Ankylosing Spondylitis Activity Index (BASDAI), Bath Ankylosing Spondylitis Metrology Index (BASMI), and fall status.
3. Physical activity and/or sedentary behavior measurement method: measurement tool, protocol, outcome measures.
4. Main results obtained from physical activity and/or sedentary behavior assessment.

## Results

The search using this strategy was completed in June 2020. We obtained a total of 23 records and 22 records after 1 duplicate was removed. A total of 12 full texts were included after screening of the abstracts and titles. Of these, 2 were then excluded after full-text screening, for a total of 10 studies included in the final review (Figure 1). Data extraction and synthesis are ongoing. Naturally, this first preliminary structured search will be repeated before the completion of the final review process. Dissemination of the study results in peer-reviewed journals is expected at the end of 2021.

## Discussion

This systematic review is designed to provide a first comprehensive and detailed synthesis of published studies that examine the use of wearable sensors for objective assessment of physical activity or sedentary behavior in patients with axSpA. The strengths of this study are its ability to directly influence future research and clinical care and the fact that this is the first time that a detailed synthesis of published studies in which wearable sensors were used for objective assessment of physical activity or sedentary behavior in patients with axSpA will be conducted. We do believe that our results could help clinicians to choose the most appropriate method to accurately monitor physical activity or sedentary behavior in this population. These results could have a major impact on diagnosis and monitoring in patients with axSpA. Furthermore, this work can guide future studies identifying preferable positions, durations, and environments. This could ultimately enable wearable device–based monitoring systems to find their way into routine clinical assessment and monitoring of patients. A major limitation of this review is the relatively small number of included studies.

The main advantage of a wearable-based monitoring approach is the passive and objective nature of the wearable data. The passive nature creates less burden for patients compared to timely manual completion of patient-reported outcomes. For clinicians, the potential benefit of wearables is related to the continuous, remote, and objective nature of the data. On the other hand, patients may be uncomfortable with continuous surveillance, and this could result in low acceptance of wearable technology despite its benefits. Furthermore, device placement errors could falsify data and lead patients and clinicians to make wrong decisions.

## Abbreviations

axSpA: axial spondyloarthritis
BASDAI: Bath Ankylosing Spondylitis Functional Index
BASFI: Bath Ankylosing Spondylitis Functional Index
BASMI: Bath Ankylosing Spondylitis Metrology Index
PEDro: Physiotherapy Evidence Database
PRISMA: Preferred Reporting Items for Systematic Reviews and Meta-Analyses
